# Regulation of TERRA on telomeric and mitochondrial functions in IPF pathogenesis

**DOI:** 10.1186/s12890-017-0516-1

**Published:** 2017-12-02

**Authors:** Yulin Gao, Jinjin Zhang, Yuxia Liu, Songzi Zhang, Youlei Wang, Bo Liu, Huizhu Liu, Rongrong Li, Changjun Lv, Xiaodong Song

**Affiliations:** 1 Department of Cellular and Genetic Medicine, School of Pharmaceutical Sciences, Binzhou Medical University, No. 346, Guanhai Road, Laishan District, Yantai City, 264003 China; 2 School of Pharmaceutical Sciences, Taishan Medical University, Taian, 271016 China; 3 Department of Respiratory Medicine, Affiliated Hospital to Binzhou Medical University, Binzhou, 256602 China

**Keywords:** IPF, lncRNA, Telomere, Mitochondria, TERRA

## Abstract

**Background:**

Aging is a known risk factor of idiopathic pulmonary fibrosis (IPF). However, the pathogenic mechanisms underlying the effects of advanced aging remain largely unknown. Telomeric repeat-containing RNA (TERRA) represents a type of long noncoding RNA. In this study, the regulatory roles of TERRA on human telomeres and mitochondria and IPF epithelial injury model were identified.

**Methods:**

Blood samples were collected from patients with IPF (*n* = 24) and matched control individuals (*n* = 24). The significance of clinical research on the TERRA expression correlated with pulmonary fibrosis was assessed. The expression levels of TERRA in vivo and in vitro were determined through quantitative real-time polymerase chain reaction analysis. Telomerase activity was observed using a fluorescent quantitative TRAP assay kit. The functions of telomeres, mitochondria, and associated genes were analyzed through RNA interference on TERRA.

**Results:**

TERRA expression levels significantly increased in the peripheral blood mononuclear cells of IPF patients. The expression levels also exhibited a direct and significantly inverse correlation with the percentage of predicted force vital capacity, which is a physiological indicator of fibrogenesis during IPF progression. This finding was confirmed in the epithelial injury model of IPF in vitro. RNA interference on TERRA expression can ameliorate the functions of telomeres; mitochondria; associated genes; components associated with telomeres, such as telomerase reverse transcriptase, telomerase, and cell nuclear antigen, cyclin D1; and mitochondria-associated cyclin E genes, including the MMP and Bcl-2 family. The RNA interference on TERRA expression can also improve the functions of oxidative-stress-associated genes, such as reactive oxygen species, superoxide dismutase, and catalase, and apoptosis-related genes, such as cytochrome c, caspase-9, and caspase-3.

**Conclusions:**

In this study, the regulation of TERRA expression on telomeres and mitochondria during IPF pathogenesis was identified for the first time. The results may provide valuable insights for the discovery of a novel biomarker or therapeutic approach for IPF treatment.

## Background

Idiopathic pulmonary fibrosis (IPF) is a specific form of chronic and progressive fibrosing interstitial pneumonia of unknown cause, and this condition occurs primarily in adults and often develops in the lungs [1]. The incidence of IPF increases remarkably with age; most patients with IPF are older than 60 years at the time of diagnosis, and young individuals are seldom affected by IPF [2]. Thus, a mechanistic link possibly exists between chronological age and this disease, although the relationship between them remains uncertain. Oxidative stress disrupts the balance between oxidant production and antioxidant defense mechanisms in tissues, and this process plays a major role in the pathogenesis of aging [3]. López-Otín et al. proposed several pivotal hallmarks, such as abnormal shortening of telomeres, epigenetic change, and mitochondrial dysfunction, which contribute to the aging process. These hallmarks can occur simultaneously and become interconnected during aging [4]. Thus, the mechanistic link among these aging hallmarks in IPF should be investigated.

Long noncoding RNAs (lncRNAs) are distinct fields of imprint in gene dosage compensation and considered by biochemists, geneticists, and computational biologists as an underlying factor in epigenetic regulation [5]. The first identified lncRNA is a gene associated with lung adenocarcinoma and designated as metastasis-associated lung adenocarcinoma transcript 1 (MALAT1) in respiratory diseases [6]. Studies on gene knockdown have shown that MALAT1 regulates the expression of genes, particularly those that do not undergo splicing and are involved in cell migration, colony formation, and metastasis [7, 8]. Our laboratory studies initially demonstrated the relationship among lncRNAs, as well as their adjacent or homologous protein-coding genes and putative miRNA-target sites. On the basis of our results, lncRNA transcription was proposed to affect the expression levels of genes adjacent to *cis* elements, hybridize to the overlapping sense transcript, act as a ceRNA, or regulate gene expression in pulmonary fibrosis [9–11]. Huang et al. [12] also supported our findings by performing motif search and manual comparisons of the data reported regarding the lungs of patients with IPF. Nevertheless, the mechanistic link between aging and lncRNA in the pathogenesis of IPF are rarely investigated.

Telomeric repeat-containing RNA (TERRA) is a type of lncRNA. Telomeres are transcribed from subtelomeric regions to large noncoding TERRA by RNA polymerase II. In the absence of regulatory mechanisms, the chromosome ends of telomeres deteriorate; these telomeres consequently become dysfunctional and thus promote DNA damage, which causes cellular senescence or rampant genome instability and apoptosis [13]. TERRA overexpression in single telomere can induce early-onset senescence [14, 15]. Telomerase activity is detected in some highly proliferative tissues, but the amount of telomerase expressed in human somatic cells is insufficient to trigger replicative senescence upon a defined number of cell divisions caused by telomere erosion. Telomerase inactivation acts as a tumor-suppressing mechanism when its re-expression in human fibroblasts facilitates the bypassing of senescence. As a result, cellular immortalization occurs. Accordingly, 90% of human cancers reactivate telomerase activity, which stabilizes telomere length [16].

Reactive oxygen species (ROS) elicit adverse effects on lung epithelial cells and fibroblasts. Fitch et al. [17] reported that lung epithelial SHH in patients with IPF increases under oxidative stress. Fernandez et al. [18] reported that ROS or hyperoxia-activated damage is an initial defense mechanism and harmful environmental stimulus in homeostatic regulation of the lung microenvironment in IPF. They also found that decreasing the oxidative load in lungs can be therapeutically beneficial. Mitochondria are well-known organelles that produce ROS and frequently function as a source of oxidants associated with oxidative stress. In the present study, the regulation of TERRA on telomeres and mitochondria in human alveolar epithelial cells of IPF was investigated. Overall, our findings may provide a diagnostic and therapeutic target to ameliorate age-associated pathologies and improve the health of patients with IPF.

## Methods

### IPF patients

IPF was diagnosed in accordance with the American Thoracic Society/European Respiratory Society consensus criteria [1], which include clinical, radiographic, and characteristic histopathological features (*n* = 24). Blood sample (5 mL) was drawn from each participant and prepared for testing. Healthy persons (*n* = 24) whose age and gender corresponded to those of the patients with IPF were then selected. Each participant provided a written informed consent. The local ethics committee approved this study.

### Animal model

C57BL/6 mice (8 weeks old) were obtained from the Model Animal Research Center of Nanjing University (Nanjing, China). All of the animal experiments were performed on the basis of the regulations established by the Ethics Committee on the Animal Experiments of Binzhou Medical University (196,738, 01/09/2014) [11]. The mice were housed under a 12 h light/dark cycle and allowed free access to food and water. The mice were then randomly divided into two groups with 10 mice in each group: sham group and bleomycin (BLM)-treated group. On day 28, all of the mice were killed, and lung tissue sections were collected and immediately frozen in liquid nitrogen for further analysis. The BLM animal model was administered with 5 mg/kg BLM dissolved in saline through single intratracheal instillation under anesthesia as previously described [9, 10].

### Cell culture

A549 (human type II alveolar epithelial cell) and MLE-12 (mouse type II alveolar epithelial cell) were purchased from the Cell Bank of the Chinese Academy of Sciences (Shanghai, China). These cells were maintained in Dulbecco’s modified Eagle’s medium for A549 and advanced minimum essential medium for MLE-12 respectively, and supplemented with 2 mM l-glutamine, 10% heat-inactivated FBS (Gibco, USA), 100 U/mL penicillin, and 100 mg/mL streptomycin.

### Cell proliferation assay

Cell proliferation was determined using a cell counting kit-8 (CCK-8; Beyotime Inst Biotech, China) in accordance with the manufacturer’s instructions. In brief, cells were seeded in a 96-well flat-bottomed plate at 1 × 10^6^ cells/well and then grown at 37 °C for 24 h. After 10 μL of WST-8 dye was added to each well, the cells were incubated at 37 °C for 2 h. Absorbance was determined at 450 nm by using a microplate reader. Cell proliferation was calculated on the basis of the coloration depth by using the following formula: cell proliferation (%) = (measurement tube absorbance − absorbance of blank) / (standard pipe of absorbance − absorbance of blank) × 100%.

### Cell growth curve analysis

An xCELLigence real-time cell analyzer (ACEA Biosciences, Inc., Hangzhou, China) was placed in an incubator in advance. Afterward, 1 × 10^5^/mL cells were seeded in a test E-Plate and placed in an analyzer, which can automatically record the cell growth curve.

### Transmission electron microscopy (TEM) observation

Cells or lung tissues were fixed with 3% fresh glutaraldehyde at 4 °C for at least 4 h and then postfixed in 1% osmium tetroxide for 1.5 h. The treated samples were dehydrated in gradient ethanol, infiltrated with Epon812, and embedded. The tissues were cultured at 37, 45, and 60 °C for 24 h. Ultrathin sections were prepared using an Ultracut E ultramicrotome. The resulting sections were stained with uranyl acetate and lead citrate and observed under a JEM-1400 TEM system (JEOL Ltd., Tokyo, Japan), as previously described [19].

### Mitochondrial membrane potential (MMP) assay

MMP was determined using 5,5′,6,6′-tetrachloro-1,1′,3,3′-tetraethylbenzimi dazolycarbocyanine iodide (JC-1; Beyotime Biotechnology, China) stain in accordance with the manufacturer’s instructions. Briefly, the harvested cells were resuspended in a mixture containing 500 μL of culture medium and 500 μL of JC-1 (5 μg/mL) staining fluid. The resulting mixture was incubated in the dark at 37 °C for 20 min. After the cells were washed twice with ice-cold staining buffer through centrifugation, the cells were resuspended in 500 μL of culture medium and then analyzed through flow cytometry. The values of MMP staining for each sample were expressed as red and green fluorescence intensities.

### Caspase activity

Caspase-3 and caspase-9 activities were obtained on the basis of the cleavage of chromogenic caspase substrates acetyl-Asp-Glu-Val-Asp *p*-nitroanilide (Ac-DEVD-pNA) and acetyl-Leu-Glu-His-Asp *p*-nitroanilide (Ac-LEHD-pNA) by using an assay kit (Beyotime Biotechnology), as previously described [20]. The cell lysate from 1 × 10^6^ cells was incubated at 37 °C for 2 h with 200 μM of Ac-DEVD-pNA (caspase-3 substrate) or Ac-LEHD-pNA (caspase-9 substrate).

The absorbance of the yellow pNA cleavage from its corresponding precursors was determined by using a spectrometer at 405 nm in a microplate reader (SpectraMax M2). The total protein concentrations in the supernatants were measured through the Bradford method.

### Apoptosis assay by flow cytometry

Suspended and adherent-treated cells (1 × 10^6^) were collected and washed with cold PBS. The fixation fluid was also washed with PBS, and 500 μL of binding buffer was added to resuspend the cells. Afterward, 5 μL of Annexin V-FITC and 5 μL of propidium iodide staining solutions were added for 20 min in the dark. Apoptosis rate was identified through flow cytometry (Beckman, Fullerton, CA, USA).

### Quantitative real-time polymerase chain reaction (qRT-PCR)

Total RNA was extracted from the blood samples/cells of the IPF patients by using TRIzol reagent from Invitrogen (Carlsbad, CA, USA) in accordance with the manufacturer’s instructions. Complementary DNA was synthesized by using M-MLV reverse transcriptase kit from Promega (Madison, WI, USA) in accordance with the manufacturer’s instructions. qRT-PCR was performed using a SYBR Green PCR Master Mix kit from Takara Bio (Shiga, Japan) on a Rotor Gene3000 real-time PCR system from Corbett Research (Sydney, Australia). The following PCR conditions were set: initial denaturation at 95 °C for 5 min, 30 cycles at 60 °C for 25 s, annealing at 52 °C for 20 s, and extension at 72 °C for 20 s. Fluorescence signal was monitored at 585 nm during each extension. Glyceraldehyde 3-phosphate dehydrogenase served as an internal control.

### ROS generation assay

Intracellular ROS levels were estimated using a kit containing the membrane-permeable fluorescent probe 2,7-dichlorofluorescin diacetate (Beyotime Biotechnology). The cells were co-activated with 5 mM of 2,7-dichlorofluorescin diacetate for 30 min. Colorimetric intensity was determined using a fluorescence spectrophotometer at excitation of 484 nm and emission of 531 nm.

### Superoxide dismutase and catalase activities

Superoxide dismutase (SOD) and catalase activities in the A549 cells were respectively determined with a total SOD assay kit and a catalase assay kit (Beyotime Biotechnology, China) following the manufacturer’s protocol [20].

### Transfection of siRNA-TERRA

The siRNAs used in our experiment were synthesized by RiboBio Co., Ltd. (Guangzhou, China). Two siRNA fragments were designed and synthesized. Afterward, 1 × 10^5^ cells were seeded in 24-well plates and cultivated with a 1640 medium containing 10% newborn calf serum for 24 h. Subsequently, 1.25 μL of 20 μM siRNA was diluted with 50 μL of 1× riboFECT™ CP buffer, and the resulting solution was incubated for 5 min at room temperature. Approximately 5 μL of riboFECT™ CP reagent was added to the solution, which was then incubated for 15 min at room temperature. The mixed solution was added to 443.75 μL of 1640 medium without 10% newborn calf serum. The cells were incubated with the mixed solution for 48 h.

### Real-time fluorescent quantitative TRAP assay

Telomerase activity was detected with a fluorescent quantitative TRAP (FQ-TRAP) kit in accordance with the manufacturer’s protocol. In brief, 1 × 10^6^ cells were lysed in 50 μL of Reagent A containing RNase inhibitors and incubated at 4 °C for 30 min. The lysate was then centrifuged at 12,000×*g* for 30 min at 4 °C, and the supernatant was collected. Protein concentration was obtained using the Bio-Rad protein reagent set. The total volume of the reaction mixture was 25 μL, which contained 15 μL of Reagent B, 1 μL of cell lysis solution, 2.5 μL of Reagent C, and 6.5 μL of Reagent D. PCR was initiated at 30 °C for 20 min, followed by 29-cycle amplification (95 °C for 20 s, 50 °C for 30 s, and 72 °C for 90 s), and terminated at 60 °C for 90 s. The telomerase activities in the cells were determined on the basis of the threshold cycle.

### Western blot analysis

The cells were collected and lysed in SDS sample buffer with protease inhibitors. Protein concentrations were determined through BCA method. First, 20 μg of proteins were separated through SDS-polyacrylamide gel electrophoresis and transferred to a polyvinylidene difluoride membrane blocked with bovine serum albumin (5%) in TBS-T. Afterward, the membrane was incubated with anti-proliferating cell nuclear antigen (anti-PCNA), anti-telomerase reverse transcriptase (anti-TERT), and anti-GAPDH antibodies at 4 °C overnight. The blots were then treated with an HRP-labeled goat antirabbit IgG (1:5000; Beijing Zhong Shan-Golden Bridge Technology Co., Ltd., Beijing, China) for 1.5 h. The membranes were subsequently washed with TBST and incubated with ECL reagent before exposure.

### Telomere length assay

After extraction of the whole genome DNA by QuickGene 610 L automatic extractor, the first qRT-PCR test was performed. The detection of telomere length primer was as follows: SEQ ID NO.1: CGGTTTGTTTGGGTTTGGGTTTGGGTTTGGGTTTGGGTT; SEQ ID NO.2: GGCTTGCCTTACCCTTACCCTTACCCTTACCCTTACCCT. The reaction system was: 4.3 μL 2× SuperReal Premix Plus, 0.3 μL SEQ ID NO.1 (10 μM), 0.3 μL SEQ ID NO.2 (10 μM), 5 μL DNA (1 ng/μL to 5 ng/μL). The total reaction volume was 10 μL. The following PCR conditions were set: initial denaturation at 98 °C for 10 min, 45 cycles at 95 °C for 30 s, and 60 °C for 1 min. Then, the second qRT-PCR test was performed. The reaction system was: 4.7 μL 2 × Taqman Expression Master Mix, 0.3 μL RNase P, 5 μL DNA (5 ng/μL). The total reaction volume was 10 μL. The following PCR conditions were set: initial denaturation at 98 °C for 10 min, 45 cycles at 95 °C for 30 s, and 60 °C for 1 min. Relative telomere length = Telomere repeat copy number/Single-gene copy number.

### Statistical evaluation

Data were statistically analyzed in SPSS version 19.0 (IBM SPSS Statistics Company, Chicago, USA). Data were presented as mean ± standard deviation of at least three independent experiments. Unpaired Student’s *t* test was used to compare the two groups. One-way ANOVA with Student–Newman–Keuls post hoc test was conducted to compare three or more groups. Statistical significance was considered at *P* < 0.05.

## Results

### Clinical research on TERRA correlation with pulmonary fibrosis

We initially assessed the significance of the correlation between clinical research on TERRA expression and pulmonary fibrosis. The characteristics and physiological processes of patients with IPF and normal persons are shown in Table 1. The number, age, and gender of the normal individuals corresponded to those of the patients with IPF. Thus, no statistical significance was observed among these parameters. PaCO_2_ was also nonsignificant. The percentage of the predicted force vital capacity (FVC%) was significant and was thus considered a physiological indicator of fibrogenesis during IPF progression [21]. Thus, this indicator was used to analyze the correlation of the TERRA expression with pulmonary fibrosis.

**Table 1:** Characteristics and physiologies of IPF patients and the normal

Characteristic	Normal	IPF	*P* value
Number	24	24	/
Age(years)	67.92 ± 7.6	69.08 ± 7.1	/
Gender (Male/female)	13/11	13/11	/
FVC (% of predicted)	89.82 ± 9.8	48.72 ± 8.1	< 0.01
TLC (% of predicted)	89.14 ± 9.7	53.47 ± 8.2	< 0.01
DLCO (% of predicted)	89.72 ± 5.9	64.05 ± 4.7	< 0.01
PaO2 (mmHg)	94.38 ± 4.9	77.34 ± 6.1	< 0.01
PaCO2 (mmHg)	39.65 ± 4.5	36.57 ± 3.8	/
Smoking History (n)	7	18	< 0.05

*FVC* forced vital capacity, *TLC* total lung capacity, *DLCO* diffusing capacity for carbon monoxide; Smoking history denotes subjects with >5 years of cigarette smoking. Two of the seven normal individuals are current smokers. No current smoker in 18 patients with IPF is observed

**Fig. 1:**
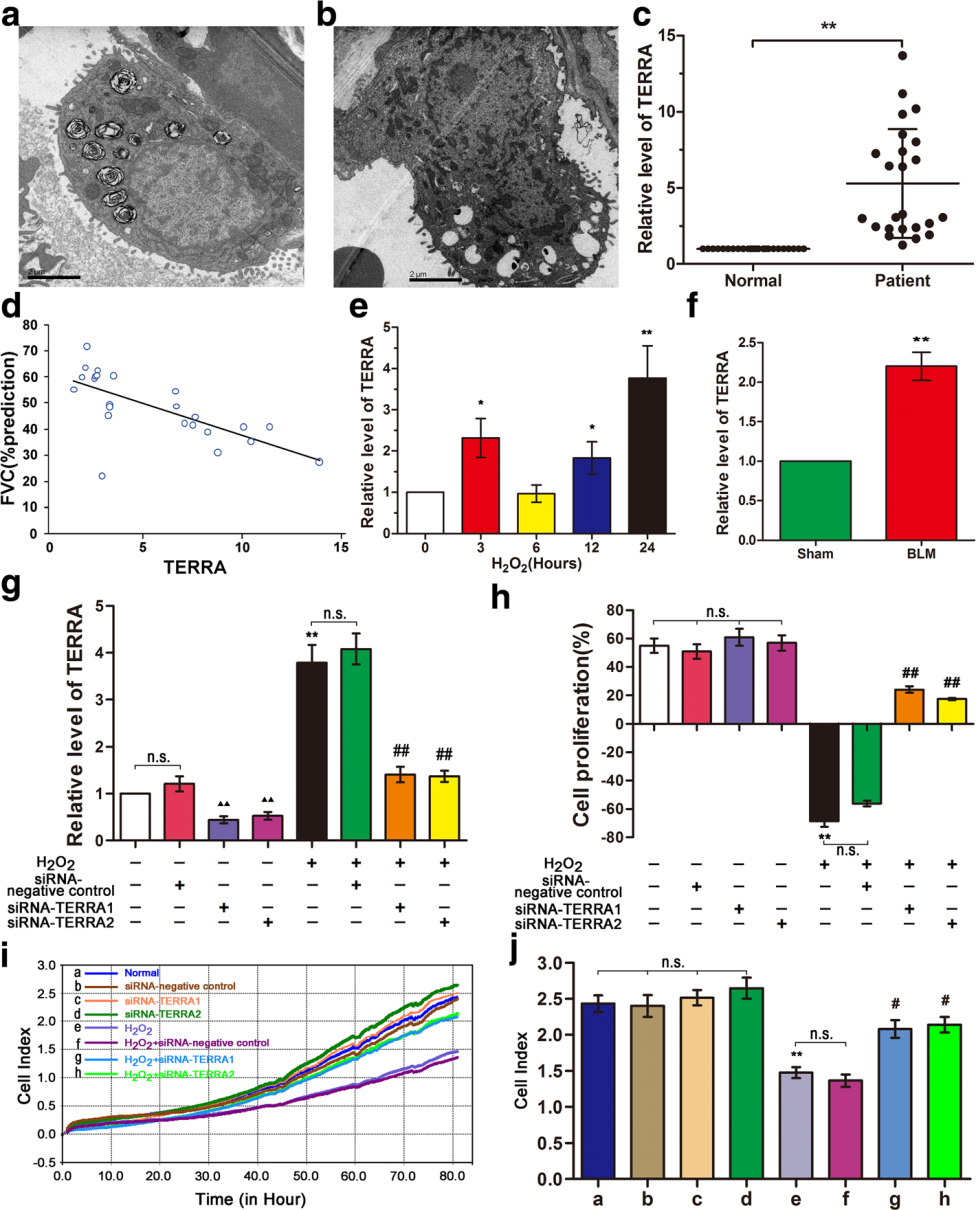
TERRA expression increased in patients with IPF and cell model. **a** AEC-II and mitochondria were observed in normal cells under TEM. Mitochondria were marked by arrows. **b** Apoptotic AEC-II and mitochondria observed in patients with IPF under TEM. **c** TERRA expression increased in the peripheral blood mononuclear cells of patients with IPF, as indicated by qRT-PCR results. The data of TERRA expression are normalized to one in every healthy person. The data in each corresponding patient are standardized by each corresponding healthy person. **d** Forced vital capacity, expressed as FVC%, significantly decreases with the TERRA expression in patients with IPF. *r* = −0.6915, *P* = 0.0002. TERRA expression was calculated by using 2^−△△Ct^. **e** TERRA expression increased with prolonged H_2_O_2_ treatment in the cell model. A549 cells were treated with 120 μM of H_2_O_2_ for 0, 3, 6, 12, and 24 h. **f** TERRA expression levels in the animal model also significantly increased. **g** The qRT-PCR data showed that siRNAs-TERRA was effectively interfered with TERRA expression in A549 cells. **h** siRNA-TERRA1 and siRNA-TERRA2 promoted the proliferation of H_2_O_2_-treated A549 cells. After transfection with siRNA-TERRA1, siRNA-TERRA2, or siRNA-negative control for 48 h, cells were co-treated with 120 μM of H_2_O_2_ for 12 h. **i** After the cells were transfected with siRNA-TERRA1, siRNA-TERRA2, or siRNA-negative control for 3 h, cells were co-treated with 120 μM of H_2_O_2_ for 3 h. Then, A549 cell growth curves were analyzed using xCELLigence real-time cell analyzer for 80 h. **j** The analytical data of the cell growth curves showed TERRA silencing can promote cell growth. Each bar represents mean ± standard deviation (SD), *n* = 6. ^*^ *P* < 0.05 and ^**^ *P* < 0.01 compared with the normal control group or sham group. ^#^ *P* < 0.05 and ^##^ *P* < 0.01 compared with the H_2_O_2_+ siRNA-TERRA negative control group. ^▲▲^ *P* < 0.01 compared with the normal + siRNA-TERRA negative control group

Type II alveolar epithelial cells (AEC-II) of lung tissues from the IPF patients were examined through TEM. The result shows that AEC-II underwent apoptosis during lung fibrosis (Fig. 1a and b). The apoptotic cells exhibited nuclear chromatin condensation along its margin, microvillus disappearance or reduction, loosening between cells, and decreased adherent ability. The shape of the cell edges became spherical. The apoptotic cells also yielded high electron density in their cytoplasms, and their organelles, except the mitochondria, were indistinguishable. This finding can be attributed to the ability of mitochondria to provide the necessary energy for apoptotic cells. Therefore, they finally disappeared during apoptosis.

The TERRA expression levels in the peripheral blood mononuclear cells collected from the IPF patients were examined through qRT-PCR. The results indicated that TERRA expression levels increased nearly fourfold in IPF patients (Fig. 1c). Notably, the TERRA expression was inversely correlated with FVC% (Fig. 1d).

Regulating TERRA expression might result in the discovery of a novel biomarker or therapeutic approach for IPF treatment. A549 and MLE-12 are appropriate and convenient in vitro models for research on AEC-II [22, 23] because of the limitations of TERRA transfection in patients or animals. Therefore, H_2_O_2_-induced A549 and MLE-12 models were established to examine the regulation of TERRA expression in telomeres and mitochondria in the epithelial injury model of IPF [24]. The cell models of IPF for A549 and MLE-12 were produced using 120 μM and 60 μM H_2_O_2_, respectively, according to previously described methods and experiments, [20, 25]. Meanwhile, qRT-PCR analysis results demonstrated that the TERRA expression levels significantly increased in the H_2_O_2_-treated A549 cell model (Fig. 1e) and further increased with prolonged H_2_O_2_ treatment. These levels also increased considerably when H_2_O_2_ treatment was performed for 3 h. The antioxidative stress initially improved the cell conditions, but the continuous application of H_2_O_2_ exceeded the antioxidative capabilities of the cells. To verify the experimental data, we analyzed the TERRA expression levels in the mouse model. We found that the TERRA expression levels also significantly increased in vivo (Fig. 1f).

In RNA interference on TERRA, A549 cells were transfected with siRNA-TERRA1 or siRNA-TERRA2 to examine the role of TERRA in the epithelial injury model of IPF. The efficiency of siRNAs-TERRA was then evaluated through qRT-PCR. siRNAs-TERRA effectively interfered with the TERRA expression in A549 cells (Fig. 1g). CCK-8 results indicated that siRNA-TERRA1 or siRNA-TERRA2 can promote A549 cell proliferation (Fig. 1h). To verify how TERRA silencing induced cell proliferation, we analyzed the cell growth curves by using a real-time cell analyzer. The analytical data showed that TERRA silencing can promote cell growth (Fig. 1i and j).

Therefore, TERRA might contribute to the pathogenesis of IPF, and RNA interference on TERRA can effectively improve this condition. Further research on the correlation of the TERRA expression with pulmonary fibrosis should be performed.

### RNA interference on TERRA can enhance the telomere function in IPF

**Fig. 2:**
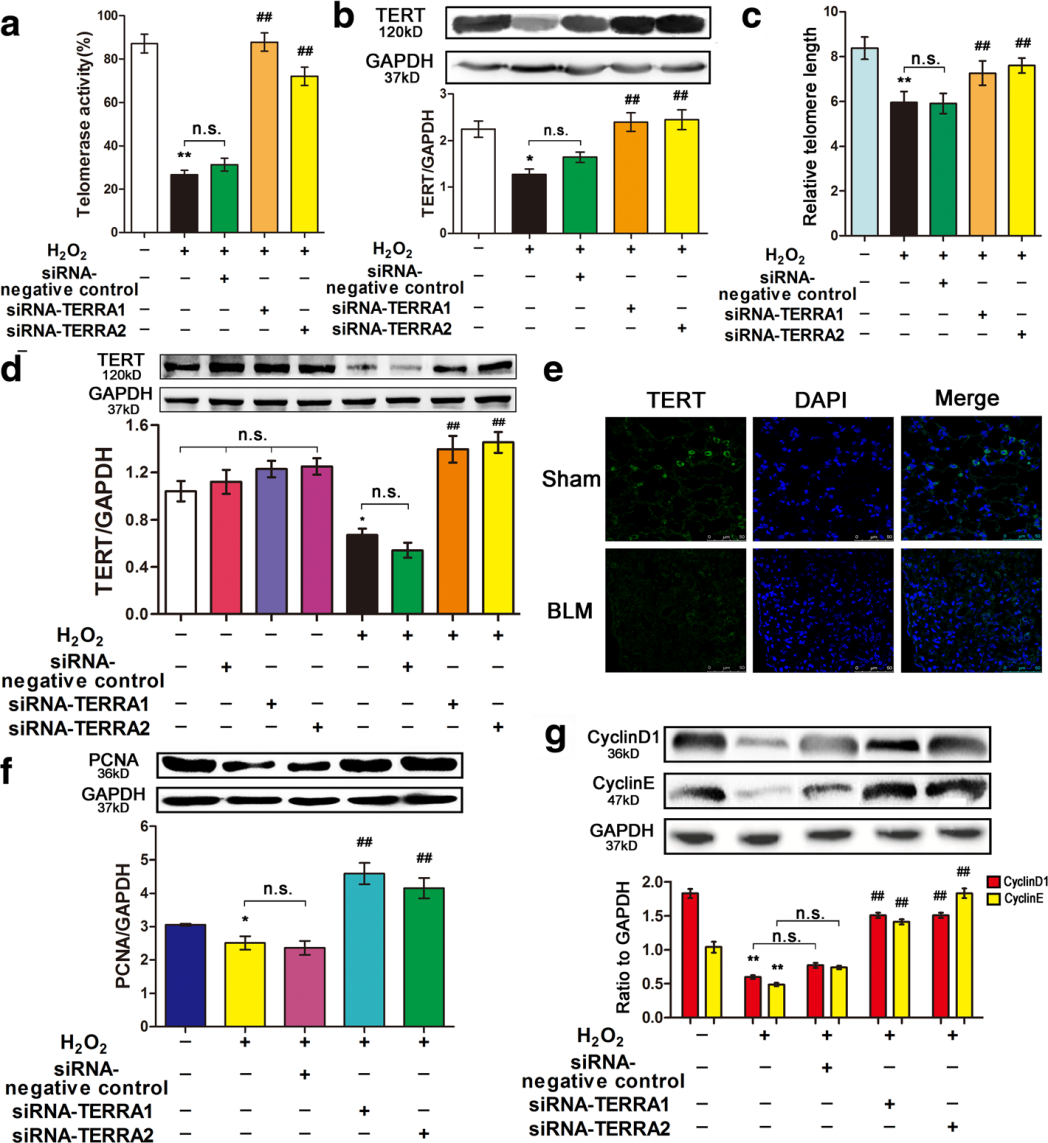
siRNA-TERRA enhanced telomere dysfunction. **a** siRNA-TERRA increased the telomerase activity in A549 cell line. **b** siRNA-TERRA promoted the TERT expression in A549 cell line. **c** The relative length of telomere was measured in A549 cells. siRNA-TERRA can promote telomere length. **d** siRNA-TERRA can also promote TERT in MLE-12 cell line. MLE-12 cells were transfected with siRNA-TERRA1, siRNA-TERRA2, or siRNA-negative control for 48 h, then co-treated with 60 μM of H_2_O_2_ for 12 h. **e** TERT expression was verified in the animal model. **f** siRNA-TERRA upregulated proliferating cell nuclear antigen expression in A549 cell line. **g** siRNA-TERRA enhanced cyclin D1 and cyclin E expression. A549 cells were transfected with siRNA-TERRA1, siRNA-TERRA2, or siRNA-negative control for 48 h and respectively co-treated with 120 μM of H_2_O_2_ for 12 h. Each bar represents mean ± SD, *n* = 6. ^*^ *P* < 0.05 and ^**^ *P* < 0.01 compared with the normal control group. ^##^ *P* < 0.01 compared with the H_2_O_2_+ siRNA-TERRA negative control group

Although telomere length corresponds to the replicative history of a cell, critically short telomeres are associated with replicative exhaustion and tissue failure. Telomerase can elongate telomeres and delay cellular aging [14]. In our study, telomerase activity was determined using the FQ-TRAP kit (Fig. 2a). Comparing with the untreated group, the researchers found that the telomerase activity significantly decreased in H_2_O_2_-treated A549 cells. Moreover, comparing with that in the H_2_O_2_-treated cells, the researchers found that the transfection with siRNA-TERRA1/2 significantly increased the telomerase activity in the A549 cells. This finding suggested that TERRA inhibited the telomerase activity. Telomerase uses telomerase reverse transcriptase (TERT) to synthesize telomere repeats [26]. Thus, TERT was analyzed through Western blot (Fig. 2b). Comparing with that in the untreated cells, the researchers found that TERT was downregulated in H_2_O_2_-treated A549 cells. Furthermore, comparing with that in the H_2_O_2_-treated cells, the researchers found that the transfection with siRNA-TERRA1/2 upregulated TERT in the A549 cells. Finally, the telomere length was measured. In siRNA-TERRA1/2 group the telomere length increased compared with that in the H_2_O_2_-treated group (Fig. 2c). To verify the data in A549 cells, we further analyzed the TERT expression in the MLE-12 cell line (Fig. 2d) and the mouse model (Fig. 2e). Therefore, TERRA inhibited TERT expression, and telomerase can elongate telomeres and delay cellular aging.

Telomerase activity is regulated by many factors, such as PCNA, cyclin D1, and cyclin E, which were analyzed by Western blot in our study (Fig. 2f and g). The trend of their expression levels was similar to that of the TERT expression. Comparing with those in the untreated ones, the researchers found that the expression levels of PCNA, cyclin D1, and cyclin E decreased in the H_2_O_2_-treated A549 cells. Moreover, comparing with those in the H_2_O_2_-treated cells, the researchers found that the expression levels of these factors in A549 cells transfected with siRNA-TERRA1/2 were increased. Therefore, RNA interference on TERRA can improve telomere dysfunction and correlated genes.

### RNA interference on TERRA can enhance the mitochondrial function in IPF

**Fig. 3:**
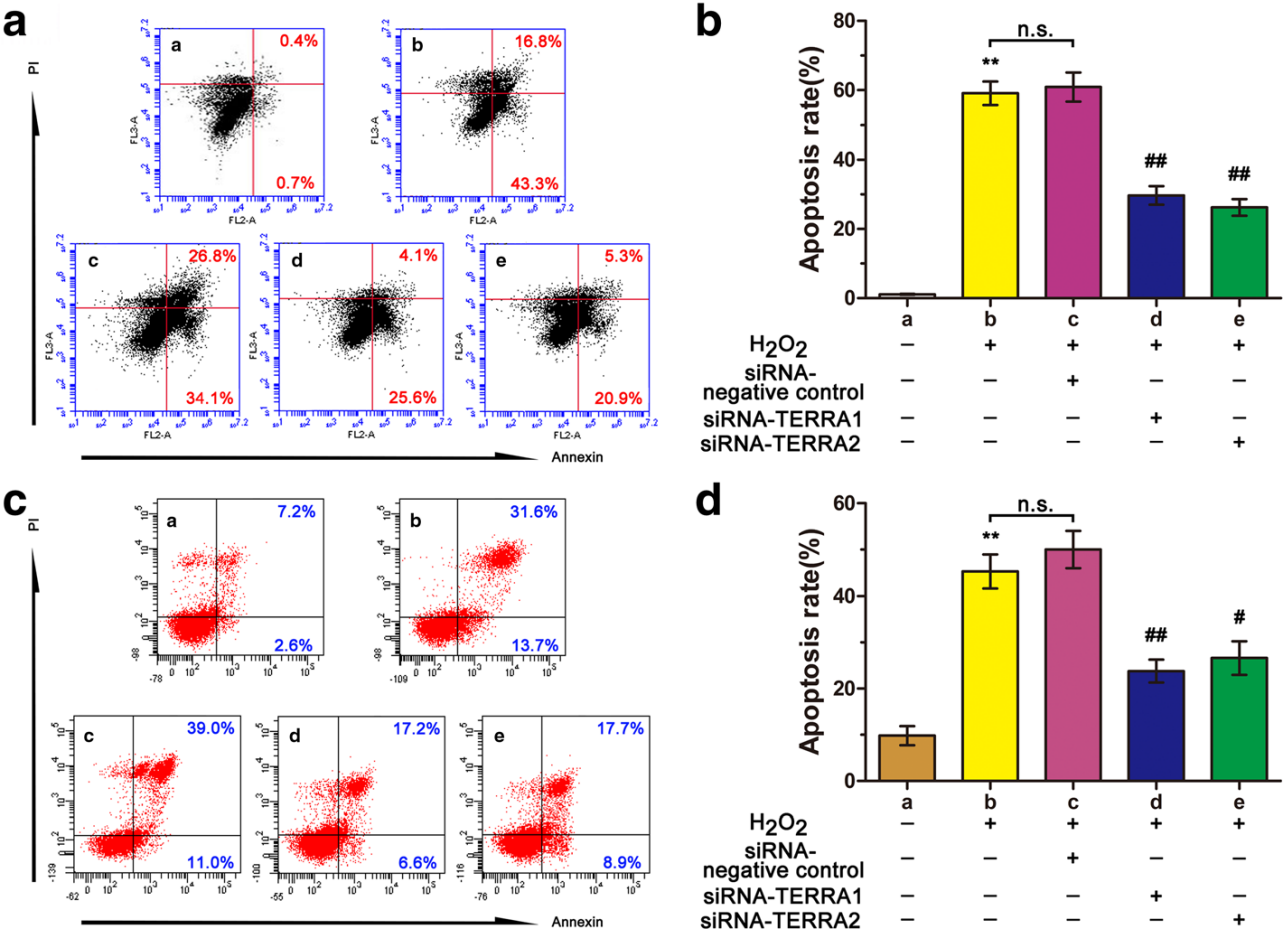
siRNA-TERRA decreased the H_2_O_2_-treated cell apoptosis. **a** Flow cytometry was used to analyze the cell apoptosis: a. normal A549 cells; b. A549 cells treated with H_2_O_2_ alone, c. A549 cells transfected with siRNA-negative control for 48 h and co-treated with 120 μM of H_2_O_2_ for 12 h, d. A549 cells transfected with siRNA-TERRA1 for 48 h and co-treated with 120 μM of H_2_O_2_ for 12 h, and e. A549 cells transfected with siRNA-TERRA2 for 48 h and co-treated with 120 μM of H_2_O_2_ for 12 h. **b** Cell apoptotic rates were quantitatively analyzed. siRNA-TERRA can decrease the apoptosis of A549 compared with H_2_O_2_-treated group. **c** The apoptosis of MLE-12 cell was tested by flow cytometry. a. normal MLE-12 cells, b. MLE-12 cells treated with H_2_O_2_ alone, c. MLE-12 cells transfected with siRNA-negative control for 48 h and co-treated with 60 μM of H_2_O_2_ for 12 h, d. MLE-12 cells transfected with siRNA-TERRA1 for 48 h and co-treated with 60 μM of H_2_O_2_ for 12 h, and e. MLE-12 cells transfected with siRNA-TERRA2 for 48 h and co-treated with 60 μM of H_2_O_2_ for 12 h. **d** Cell apoptotic rates were quantitatively analyzed. siRNA-TERRA can decreased the apoptosis of MLE-12 compared with H_2_O_2_-treated group. Each bar represents mean ± SD, *n* = 6. ^**^ *P* < 0.01 compared with the normal control group. ^#^ *P* < 0.05 and ^##^ *P* < 0.01 compared with the H_2_O_2_+ siRNA-TERRA negative control group

**Fig. 4:**
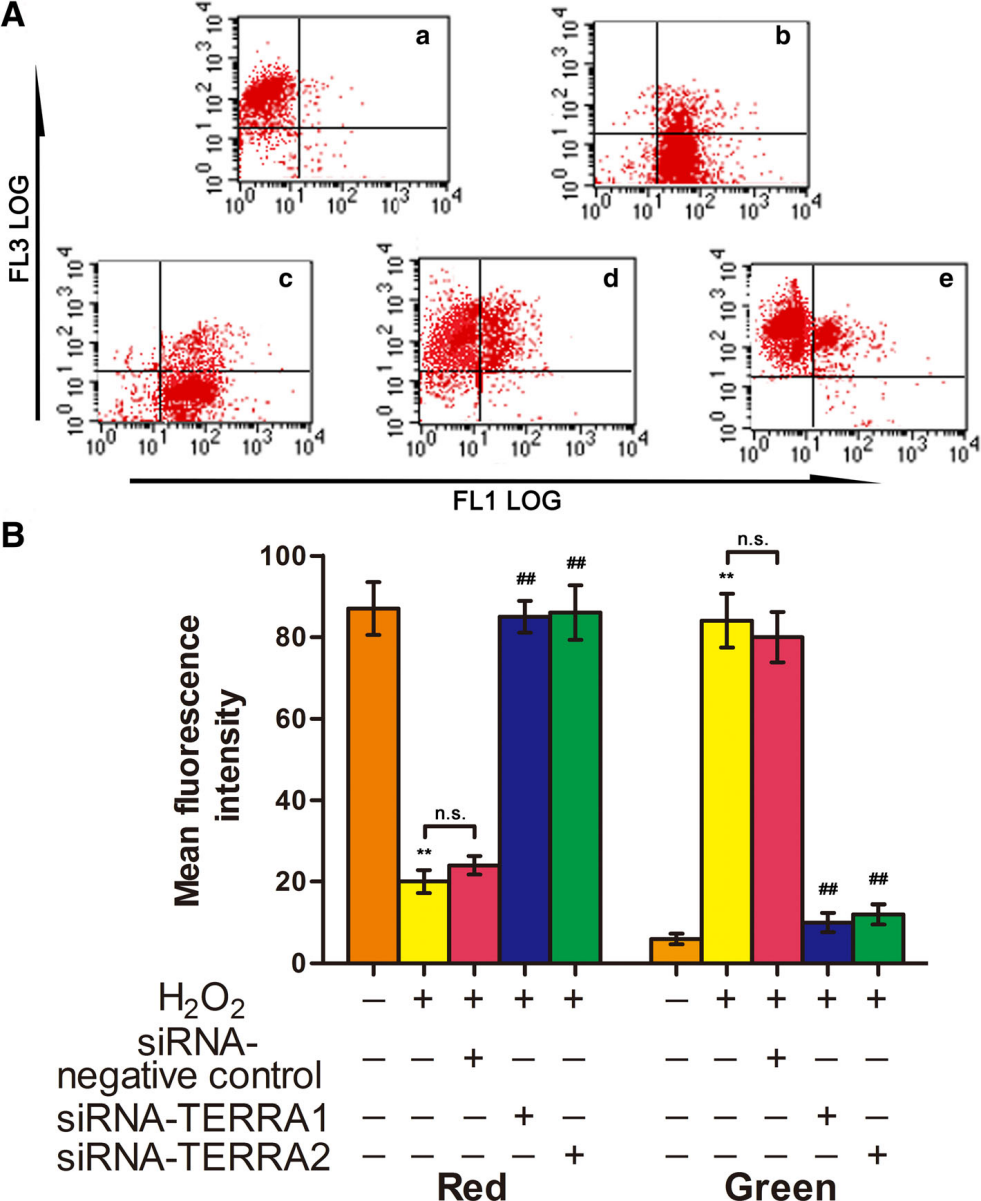
siRNA-TERRA increased MMP in cells treated with H_2_O_2_. **a** Flow cytometry was used to analyze MMP: a. normal A549 cells, b. A549 cells treated with 120 μM of H_2_O_2_ alone for 12 h, c. A549 cells transfected with siRNA-negative control for 48 h and co-treated with 120 μM of H_2_O_2_ for 12 h, d. A549 cells transfected with siRNA-TERRA1 for 48 h and co-treated with 120 μM of H_2_O_2_ for 12 h, and e. A549 cells transfected with siRNA-TERRA2 for 48 h and co-treated with 120 μM of H_2_O_2_ for 12 h. **b** MMP results were quantitatively analyzed. Each bar represents mean ± SD, *n* = 6. ^**^ *P* < 0.01 compared with the normal control group. ^##^ *P* < 0.01 compared with the H_2_O_2_+ siRNA-TERRA negative control group

The apoptosis of AEC-II is a critical event that initiates and propagates fibrosis in the lung parenchyma. The mitochondria are the control center of apoptosis and oxidative stress. Thus, flow cytometry was conducted to quantitatively analyze cell apoptosis. The results showed that cell apoptosis increased by 59.10% when the A549 cells were treated with H_2_O_2_ alone. Nevertheless, these apoptotic rates were decreased to 29.1% and 26.2% by siRNA-TERRA1 and siRNA-TERRA2, respectively (Fig. 3a and b). To verify the apoptotic rate in the A549 cells, we evaluated the apoptosis of the MLE-12 cell line. The apoptosis of MLE-12 cells was also decreased by siRNA-TERRA (Fig. 3c and d). MMP was also determined by JC-1 staining through flow cytometry. The MMP results (Fig. 4) revealed that H_2_O_2_ reduced the MMP in the A549 cells, as indicated by the decreased red cells and increased green cells. Moreover, siRNA-TERRA can increase the MMP expression in the H_2_O_2_-treated cells. Therefore, RNA interference on TERRA can disrupt AEC-II apoptosis and MMP disintegration.

### RNA interference on TERRA can contribute to antioxidative stress

**Fig. 5:**
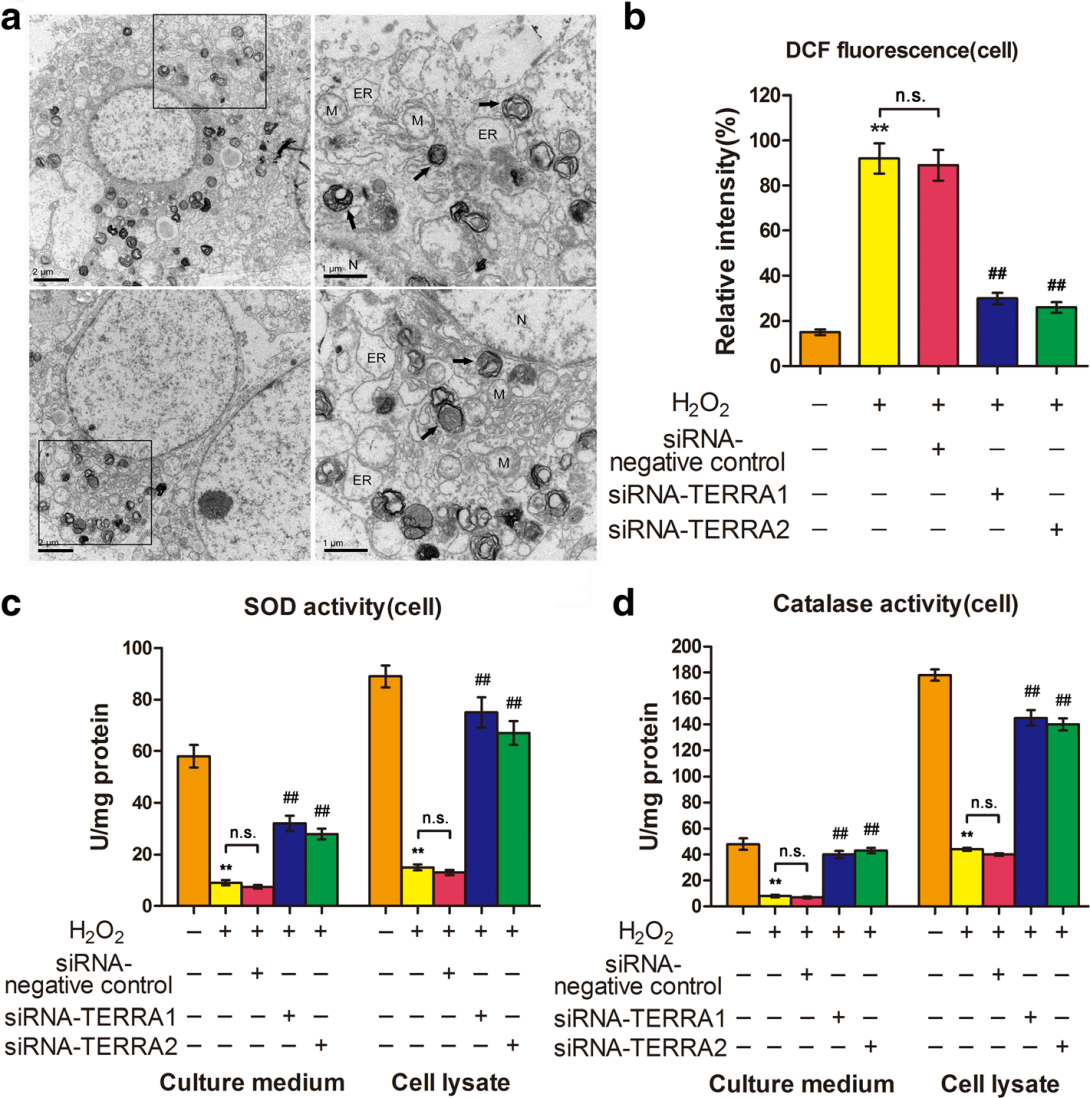
Antioxidative properties of siRNA-TERRA facilitated the protection against oxidative stress. **a** Mitochondria and autophagy were observed in A549 cells treated with 120 μM of H_2_O_2_ alone for 12 h using TEM. Mitochondria are marked by M. Autophagy is marked by an arrow. Endoplasmic reticulum is marked by ER. **b** ROS generation was determined using 5 μM of CMH2DCFDA after treatment. siRNA-TERRA significantly decreased ROS generation in A549 cells. **c** siRNA-TERRA significantly increased superoxide dismutase activities. **d** siRNA-TERRA significantly increased catalase activities. Each bar represents mean ± SD, *n* = 6. ^**^ *P* < 0.01 compared with the normal control group. ^##^ *P* < 0.01 compared with the H_2_O_2_+ siRNA-TERRA negative control group

Oxidative stress may contribute to mitochondrial structural damage and autophagy. TEM results showed that some of the mitochondria of the H_2_O_2_-treated A549 cells were swollen and contain vacuole-like structures. The cells also contain organelles with few cristae or deformed cristae and mitochondria with cristae parallel to the long axis. Furthermore, a high degree of autophagy was observed in H_2_O_2_-treated A549 cells (Fig. 5a).

**Fig. 6:**
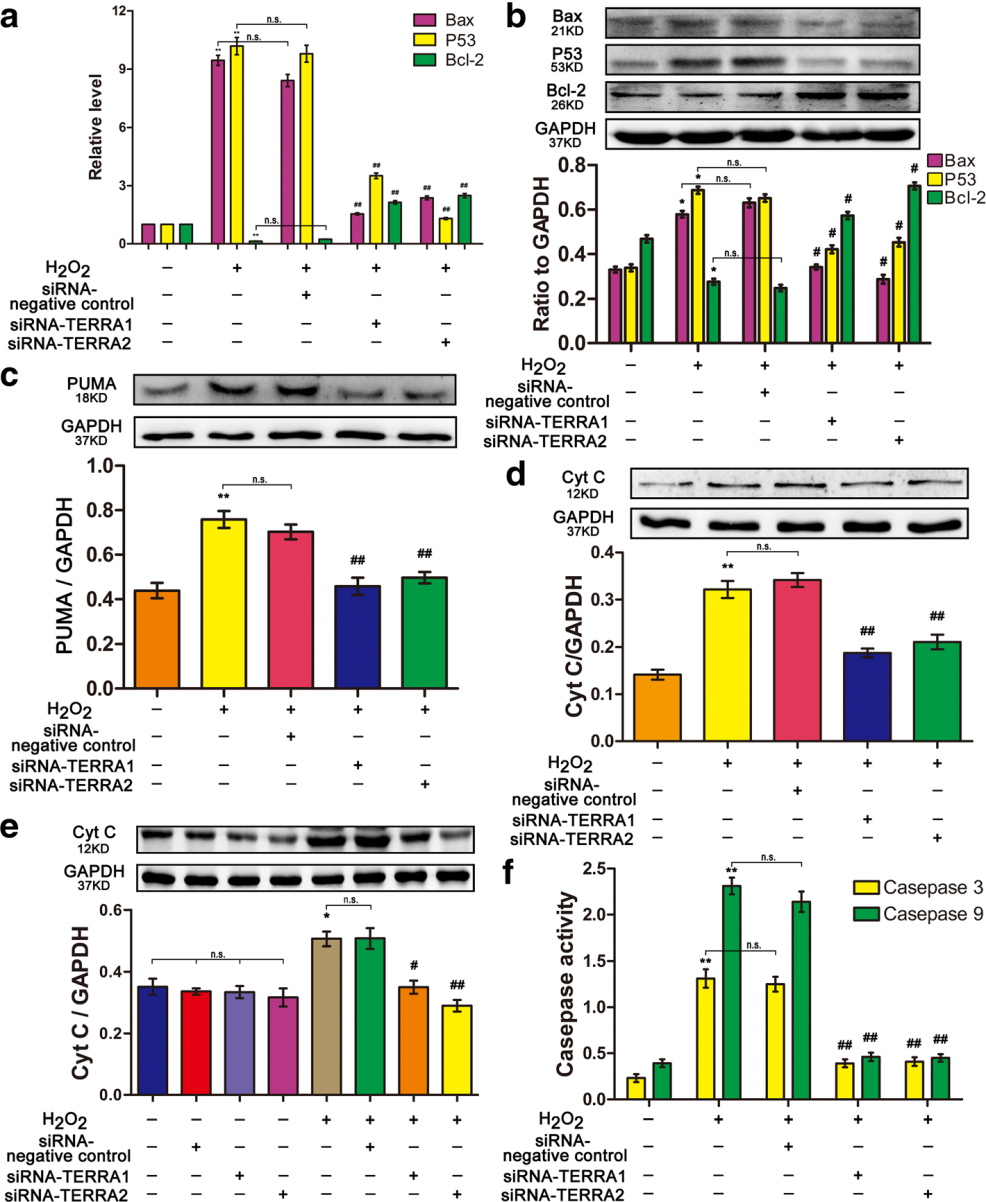
siRNA-TERRA changed the associated gene expression. **a** p53, bax, and Bcl-2 were analyzed by qRT-PCR in A549. siRNA-TERRA-treated cells exhibited reduced activities of p53 and bax but increased Bcl-2 at mRNA level. **b** p53, bax, and Bcl-2 were analyzed by Western blot in A549. siRNA-TERRA-treated cells possessed decreased activities of p53 and bax but increased Bcl-2 at protein levels. **c** The activities of puma and (**d**) cytc were reduced in siRNA-TERRA-treated A549 cells. **e** Activity of cytc was also reduced in siRNA-TERRA-treated MLE-12 cells. **f** Caspase-3 and caspase-9 decreased markedly in siRNA-TERRA-treated A549 cells. Each bar represents mean ± SD, *n* = 6, ^*^ *P* < 0.05 and ^**^ *P* < 0.01 compared with the normal group; ^#^ *P* < 0.05 and ^##^ *P* < 0.01 compared with the H_2_O_2_+ siRNA-TERRA negative control group

ROS, SOD, and catalase were examined. We determined the ROS production in A549 cells under different conditions to determine the effects of TERRA or siRNA-TERRA on H_2_O_2_-induced ROS production (Fig. 5b). The ROS produced in the H_2_O_2_-treated cells was highly expressed but was suppressed by siRNA-TERRA1 or siRNA-TERRA2. We hypothesized that H_2_O_2_ generated ROS by inhibiting antioxidant enzymes, such as SOD and catalase, whereas siRNA-TERRA suppressed H_2_O_2_-induced ROS generation by protecting the antioxidant enzymes from H_2_O_2_. The cells with and without siRNA-TERRA were treated with H_2_O_2_ to confirm this hypothesis. SOD (Fig. 5c) and catalase activities (Fig. 5d) were determined. The decrease in SOD and catalase activities after H_2_O_2_ treatment is shown in Fig. 6b and c, respectively. The inhibitory effect of H_2_O_2_ on SOD and catalase activities suggested that the cytotoxic effect of H_2_O_2_ was caused by the oxidative stress in the cells, but siRNA-TERRA can protect these antioxidant enzymes from H_2_O_2_. Therefore, siRNA-TERRA might act as an inhibitor (antioxidant) to H_2_O_2_-mediated ROS generation and help protect cells from oxidative stress during the development of IPF.

### RNA interference on TERRA can change senescence-regulatory and apoptotic genes

**Fig. 7:**
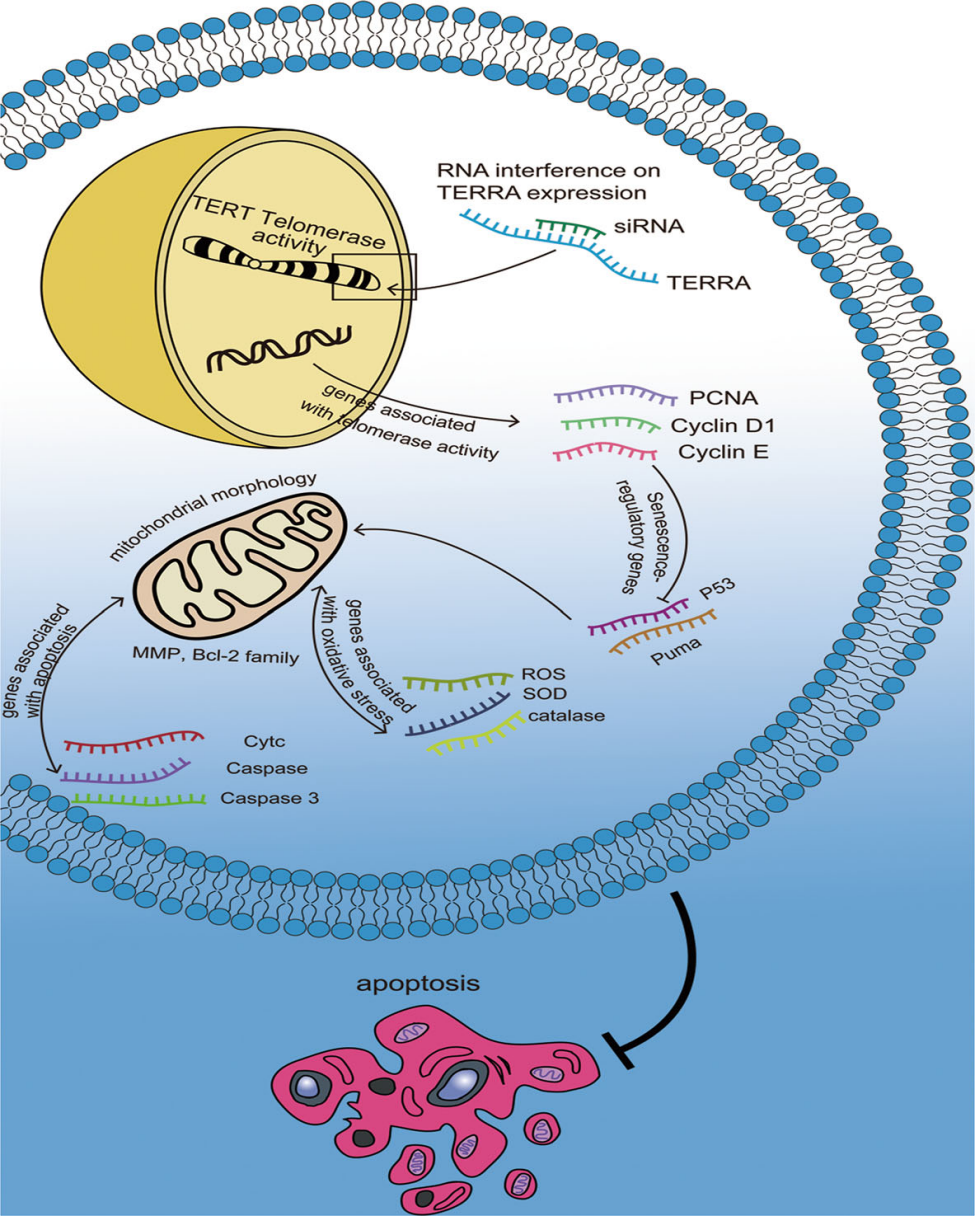
RNA interference on TERRA can improve the telomeric and mitochondrial functions via the major senescence-regulatory pathway of p53 in IPF pathogenesis

Telomeric and mitochondrial dysfunctions can influence apoptotic gene expression. In this study, the senescence-regulatory genes of p53- and p53-upregulated modulator of apoptosis (puma) were also tested. Tumor protein p53, puma, apoptosis regulator bax, Bcl-2, and cytc were analyzed by qRT-PCR or Western blot (Fig. 6a–e). In addition, caspase-3 and caspase-9 were evaluated by using a microplate reader (Fig. 6f). The results showed that p53, puma, bax, cytc, caspase-3, and caspase-9 increased markedly in the cells treated with H_2_O_2_ alone. The activities of p53, puma, bax, cytc, caspase-3, and caspase-9 in the siRNA-TERRA-treated cells were reduced in contrast to those in the H_2_O_2_-treated cells. The anti-apoptotic Bcl-2 exhibited an opposite trend. Its expression level decreased in the cells treated with H_2_O_2_ alone but increased in the cells co-treated with siRNA-TERRA. Thus, telomeric and mitochondrial functions were impaired. The major senescence-regulatory pathway of p53 was activated in IPF, and RNA interference on TERRA can improve this condition (Fig. 7).

## Discussion

Many advances enhanced our understanding of IPF pathogenesis and presented substantial evidence supporting several aging mechanisms, including oxidative stress, telomere length regulation, mitochondrial dysfunction, and changes in the number of anti-aging molecules in IPF [27]. A further understanding of these abnormalities may help design and improve novel therapeutic interventions for IPF patients. In particular, lncRNA is considered essential for various molecular and cellular processes, such as senescence, proliferation, apoptosis, and differentiation. Therefore, the influence of lncRNA on cellular and molecular bases of aging was reviewed [28]. However, the underlying mechanisms of lncRNAs in IPF remain poorly understood. The regulation of lncRNA and its interference should be investigated to explain the pathogenesis of IPF and provide a biomarker or therapeutic target for IPF patients. In the present work, the TERRA expression levels in the peripheral blood mononuclear cells from IPF patients were inversely correlated with FVC%. This finding is considerably important because lung biopsy is the only method that can accurately diagnose IPF. However, lung biopsy is an invasive procedure with high morbidity and mortality risks, and it may be unsuitable for some individuals. Determining an appropriate biomarker in the peripheral blood of IPF can provide a useful noninvasive method for IPF treatment. Thus, regulating the TERRA expression level can provide a novel biomarker or therapeutic approach for IPF treatment. In our study, a cell model was used to demonstrate that TERRA might contribute to the IPF pathogenesis, and RNA interference on TERRA can improve telomeric and mitochondrial functions (Fig. 7).

Oxidative stress plays a key role in aging because oxidative changes can provide mechanistic switches that control protein conformation, catalytic activity, protein–protein interaction, protein–DNA interaction, and protein trafficking [29, 30]. IPF Patients possess increased markers of oxidative stress locally in their lungs and systemically in their bodies [31, 32]. As a marker of oxidative stress, ROS promotes the occurrence of pulmonary fibrosis by activating the crucial mediators of epithelial–mesenchymal transition. Protection from free radicals is attained through different mechanisms, such as SOD and catalase, which are enzymatic systems that decompose superoxide radicals to H_2_O_2_. As expected, the increased ROS generation in the H_2_O_2_-induced lung fibrosis models was more evident than that in the other groups. siRNA-TERRA can also suppress ROS generation. Evidently, the activities of SOD and catalase decreased in the H_2_O_2_-induced lung fibrosis models, and siRNA-TERRA promoted SOD and catalase activities. Accordingly, the dysregulation of lncRNA expression plays a key role in cellular stress responses [33, 34]. Our findings indicated that TERRA can facilitate IPF pathogenesis.

Telomeres protect cells, facilitate cell proliferation, and enable DNA polymerase to complete replication. The length of telomere corresponds to the length at birth and rate of attrition thereafter. The latter indicates several factors, such as cumulative oxidative stress [35], which acts on progenitor cells. Thus, replicative senescence is elicited when the telomere length is decreased substantially. Accordingly, replicative senescence can be delayed by restoring telomerase expression, which replenishes telomeres. Critically short telomeres initiate the major senescence-regulatory pathway of p53; consequently, normal activity is reduced and age-related diseases are exacerbated [36]. In particular, genetic and clinical evidence indicates that short telomere phenotype appears in most IPF patients [37, 38]. Short telomeres in lung epithelial cells and peripheral blood cells are also observed in patients with IPF [39]. Remarkably, 10% of patients with familial pulmonary fibrosis experience mutations in TERT, which is a key factor in telomere elongation [40]. TERRAs also binds telomerase core components, TERC and TERT, but their role in telomerase function is unclear. On the one hand, TERRA can inhibit telomerase activity in vitro. On the other hand, in yeast, TERRAs are induced at short telomeres and form TERRA-TERC RNA clusters [41]. Bleomycin causes an initial increase, and then a reduction, in telomerase activity controlled, at least in part, at the mRNA level. When telomerase activity is diminished, significant apoptosis of epithelial cells is initiated. With further disruption of telomerase activity, apoptosis occurs at significantly higher levels [42].

We demonstrated that TERRA can inhibit telomerase activity and TERT expression to prevent telomere elongation. Consequently, TERRA can prevent the expression of PCNA, cyclin D1, and cyclin E. Our findings were consistent with previous results, which showed that telomeres are reduced during cell cycle when DNA polymerase is used because the priming of DNA synthesis does not readily occur in this region [43, 44]. In our study, p53 was activated by H_2_O_2_, but its expression was inhibited when TERRA was interrupted by siRNA. On the basis of these results, we inferred that increased TERRA expression levels are associated with telomere dysfunctions caused by oxidative stress, and this phenomenon contributes to IPF pathogenesis.

Lungs are among the organs most exposed to various forms of ROS because of their high-oxygen-containing environment. The respiratory chain in the mitochondria is a significant endogenous source of ROS. Thus, the protective role of mitochondria in pulmonary fibrosis is closely associated with their function in ROS balance maintenance [24]. Sosulski et al. [45] proposed that enhancing the autophagic flux and mitochondrial recycling through hormetic compounds can diminish the expression of damaging ROS, maintain mitochondrial functions, normalize lung fibroblast phenotype, and promote a healthy lung. In the present study, the lung epithelial cells exhibited a substantial accumulation of dysmorphic and dysfunctional mitochondria, which supported our hypothesis. We also evaluated the cell apoptotic rate, changes in mitochondrial morphology, MMP, and electron transport chain. Dysfunctional mitochondria promoted cell apoptosis, caused structural integrity failure, decreased MMP, and damaged the electron transport chain under oxidative stress. These adverse conditions were mitigated to varying degrees when TERRA was inhibited by siRNA. Mitochondria are particularly susceptible to aging, and related abnormalities often include enlargement, loss of cristae, destruction of inner membranes, swelling, and impaired respiration. Bueno et al. [46] evaluated the mitochondria in the lung epithelial cells of human patients with IPF and proposed that dysfunctional mitochondria promotes fibrosis in an aging lung.

Despite extensive research efforts over the past decades, effective therapies that uses corticosteroids, azathioprine, and cyclophosphamide for IPF are unavailable; thus, lung transplantation is currently the only effective therapy [47, 48]. Alternative approaches and novel targets and pathways in IPF are urgently necessary to overcome poor prognosis and lack of available therapies. Although miR-122 is unrelated to IPF, targeting liver-specific miR-122 through an antisense-based approach is currently undergoing human trials to treat hepatitis C virus [49]. Therefore, the cellular and molecular mechanisms of lncRNA and its antisense on IPF should be examined. Considering the trait of lncRNA, we should further explore new fields for IPF treatment.

## Conclusions

The identification of the regulatory function of TERRA can provide a diagnostic or therapeutic target to ameliorate age-associated pathologies and improve the health of patients with IPF.

## Abbreviations

CCK-8: Cell counting kit-8
DLCO: Diffusing capacity for carbon monoxide
FQ-TRAP: Real-time fluorescent quantitative TRAP assay
FVC: Forced vital capacity
IPF: Idiopathic pulmonary fibrosis
LncRNA: Long noncoding RNA
MMP: Mitochondrial membrane potential
PCNA: Proliferating cell nuclear antigen
qRT-PCR: Quantitative real-time polymerase chain reaction
ROS: Reactive oxygen species
SOD: Superoxide dismutase
TEM: Transmission electron microscopy
TERRA: Telomeric repeat-containing RNA
TERT: Telomerase reverse transcriptase
TLC: Total lung capacity
